# Internalizing the threat of risk-a qualitative study about adolescents’ experience living with screening-detected celiac disease 5 years after diagnosis

**DOI:** 10.1186/1477-7525-12-91

**Published:** 2014-06-11

**Authors:** Katrina Nordyke, Anna Rosén, Maria Emmelin, Anneli Ivarsson

**Affiliations:** 1 Department of Public Health and Clinical Medicine, Epidemiology and Global Health, Umeå University, Umeå, Sweden; 2 Department of Clinical Sciences, Social Medicine and Global Health, Lund University, Lund, Sweden

**Keywords:** Adolescents, Celiac disease, Gluten-free diet, Narratives, Qualitative research, Screening

## Abstract

**Background:**

Mass screening could identify those with unrecognized celiac disease (CD), but the experience of being detected through screening and living with screening-detected CD should be explored before considering this as acceptable intervention. For this study we invited screening-detected adolescents to describe their experience living with screening-detected CD five years after diagnosis with the aim to explore how their perceptions, practices, and beliefs evolved.

**Methods:**

Adolescents who were diagnosed through a population-based CD screening were invited to write narratives after being diagnosed. Of 153 adolescents who were eventually diagnosed through the screening, 91 wrote narratives one year after diagnosis and 72 five years after diagnosis. A qualitative content analysis resulted in a theme and categories that describe the experience living with screening-detected CD five years after diagnosis.

**Results:**

The overall theme **–*Internalizing the threat of risk*–** illustrates that being detected through screening and the internalized threat of future health complications have impacted how these adolescents felt about the diagnosis, coped with the gluten-free diet (GFD), and thought about CD screening. This theme is supported by four categories: **maintaining an imposed disease identity** describes how they continued to define their diagnosis in relation to the screening. They also expressed **moving from forced food changes to adapted diet routines** by describing habits, routines, coping strategies, and the financial burden of the GFD. They had **enduring beliefs of being spared negative consequences,** however, even after five years, some doubted they had CD and worried that being detected and eating a GFD might not be beneficial, i.e. **continuing to fear it is “all in vain”**.

**Conclusions:**

There was maintenance and evolution in the perceptions, practices, and beliefs of the adolescents after five years. Some have adjusted to the disease and adapted new habits and coping strategies to deal with the GFD, while others still doubt they have CD or that being detected was beneficial. The transition to adapting to the disease and GFD is ongoing, illustrating the importance of providing ongoing support for those with screening-detected CD as they adjust to this chronic disease and the GFD.

## Background

Celiac disease (CD) is a chronic autoimmune disorder in genetically predisposed individuals in which damage to the small intestine is caused by eating foods containing gluten (found in wheat, rye, and barley) [1]. Serologic markers indicative of CD are highly predictive [2,3] and diagnosis is based on a biopsy of the small intestine revealing enteropathy [4,5]. The treatment is a lifelong gluten-free diet (GFD) and in most patients who adhere to the diet the enteropathy and symptoms resolve [4,6]. Health consequences of untreated CD can be related to malabsorption, caused by the intestinal enteropathy, or systemic, related to the body’s immunologic response to the inflammation of the gut [1,7]. Complications of untreated CD include, but are not limited to, diarrhea, constipation, stomachache, fatigue, delayed puberty, anemia, depression, and low bone mineral density [4,5,7-9]. However, signs and symptoms can also be subtle, absent, or not recognized as CD-related and this can make it difficult to detect in routine clinical practice [9,10]. Although the prevalence is generally suggested to be around 1%, most people with CD are undiagnosed [11-14]. Recently, a Swedish CD screening study found a prevalence of 3% with 2/3 previously undetected [15,16]. How to go about finding those with unrecognized CD is debated, one option includes mass screening [9,10,17-19].

Mass screening, sometimes referred to as population screening, may be an option for identifying those with unrecognized CD [9]. Anthony Giddens has elaborated on modern social theory including the issue that individuals still feel at risk even in what seems to be an increasingly controlled environment [20]. Sometimes it may seem that a goal of modern society is to create a “zero-risk society” but even attempts to decrease risk introduce the potential for new risks [20,21]. Screening involves actively seeking to identify a pre-disease or disease condition in people who are presumed healthy [22]. It can be assumed that those who consider themselves healthy, but receive a chronic disease diagnosis as a result of screening, have a different experience than those who seek care because of symptoms [22]. If individuals who consider themselves healthy are approached to participate in a screening, the benefit from being detected and treated must outweigh any harm caused through the screening [23,24]. This includes not only the physical benefit and harm, but should also take into account how their quality of life is affected.

Health-Related Quality of Life (HRQoL) includes physical, psychological, and social domains of health as influenced by a person’s experiences, beliefs, expectations, and perceptions [25]. Research addressing the HRQoL and Quality of Life (QoL) of people with CD is based mostly on those diagnosed through routine clinical practice or selectively screened because they are considered at high risk [26-40]. Only a few studies have addressed the HRQOL/QOL of children or adolescents who were diagnosed through population screening [41-43]. In a 10-year follow-up study, children who were screening-detected and adhered to a GFD had a HRQoL similar to that of the general population [41]. The follow-up studies we have previously reported on revealed a complex situation for screening-detected adolescents. We have shown that screening-detected adolescents reported a similar HRQoL before and one year after diagnosis (and similar to their peers without CD) [42]. We also have reported findings from screening-detected adolescents who answered a questionnaire (n = 93) one year after diagnosis in which 54% reported feeling better, 4% worse, 37% no difference, and 5% do not remember how they felt before diagnosis [43].

For this present study we invited screening-detected adolescents to describe their experience living with the screening-detected disease five years after diagnosis to explore how their perceptions, practices, and beliefs have evolved.

## Methods

### Study setting

The adolescents participated in a population-based CD screening study. The study, known as ETICS (*Exploring The Iceberg of Celiacs in Sweden*), took place in five different regions in Sweden and 10,041 sixth graders (12-year-olds) were invited to participate [15]. Blood samples were collected from 7,208 (72%) children (without a previous CD diagnosis), and were analyzed for CD serological markers [15]. Children with elevated markers were referred for an intestinal biopsy used for a definitive diagnosis [15,44]. For those with a confirmed CD diagnosis, follow-up care was provided according to current clinical standards including recommending a GFD and dietician support. In addition, as part of the ETICS study, these children were also invited to follow-up studies one- and five-years after the screening which is not a routine part of follow-up after clinical diagnosis. The follow-up studies included questionnaires, narrative submissions, and focus group discussions. Information was provided to parents and adolescents, written consent was obtained from parent/s for all participating adolescents, and withdraw at any time was allowed. Ethical approval for the study was granted by the Regional Ethical Review Board at Umeå University, Sweden.

### Study design

This study utilized a qualitative approach based on narratives written by the adolescents. Written narratives can give insight into people’s own perspectives, interpretations, and an understanding of meanings they attach to experiences [45]. For the one-year follow-up adolescents were asked to write at home and mail in their narrative. For the five-year follow-up they were asked to write their narrative during their clinical visit and return it directly to the doctor. On both occasions they were encouraged to write in whatever manner they wanted, but to address: *How it felt to find out they had CD, If life became different after they found out, How they thought it worked with food at home and at school,* and *If they thought it would be good to test all children for CD and if so at what age*.

### Participants

**Figure 1:**
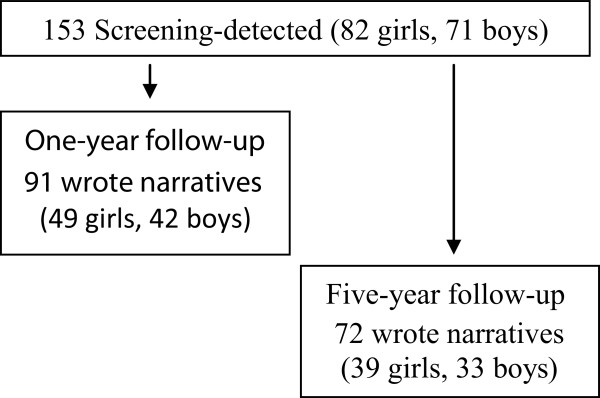
**Adolescents with screening-detected celiac disease wrote narratives one and five years after diagnosis.** Detailed legend: Of the 153 adolescents who ultimately received a screening-detected celiac disease diagnosis, 91 wrote narratives about one year after diagnosis and 72 wrote narratives about five years after diagnosis.

Of the 153 (82 girls, 71 boys) adolescents who eventually received a screening-detected CD diagnosis [15,16,44], 91 (49 girls, 42 boys) wrote narratives about one year after diagnosis (median age 14.6) and 72 (39 girls, 33 boys) wrote narratives about five years after diagnosis (median age 18.0) (Figure 1). There were 43 who wrote narratives on both occasions. The distribution of the sexes in the one- and five-year follow-up is similar with girls accounting for 54% in each group (Figure 1).

### Analysis

**Figure 2:**
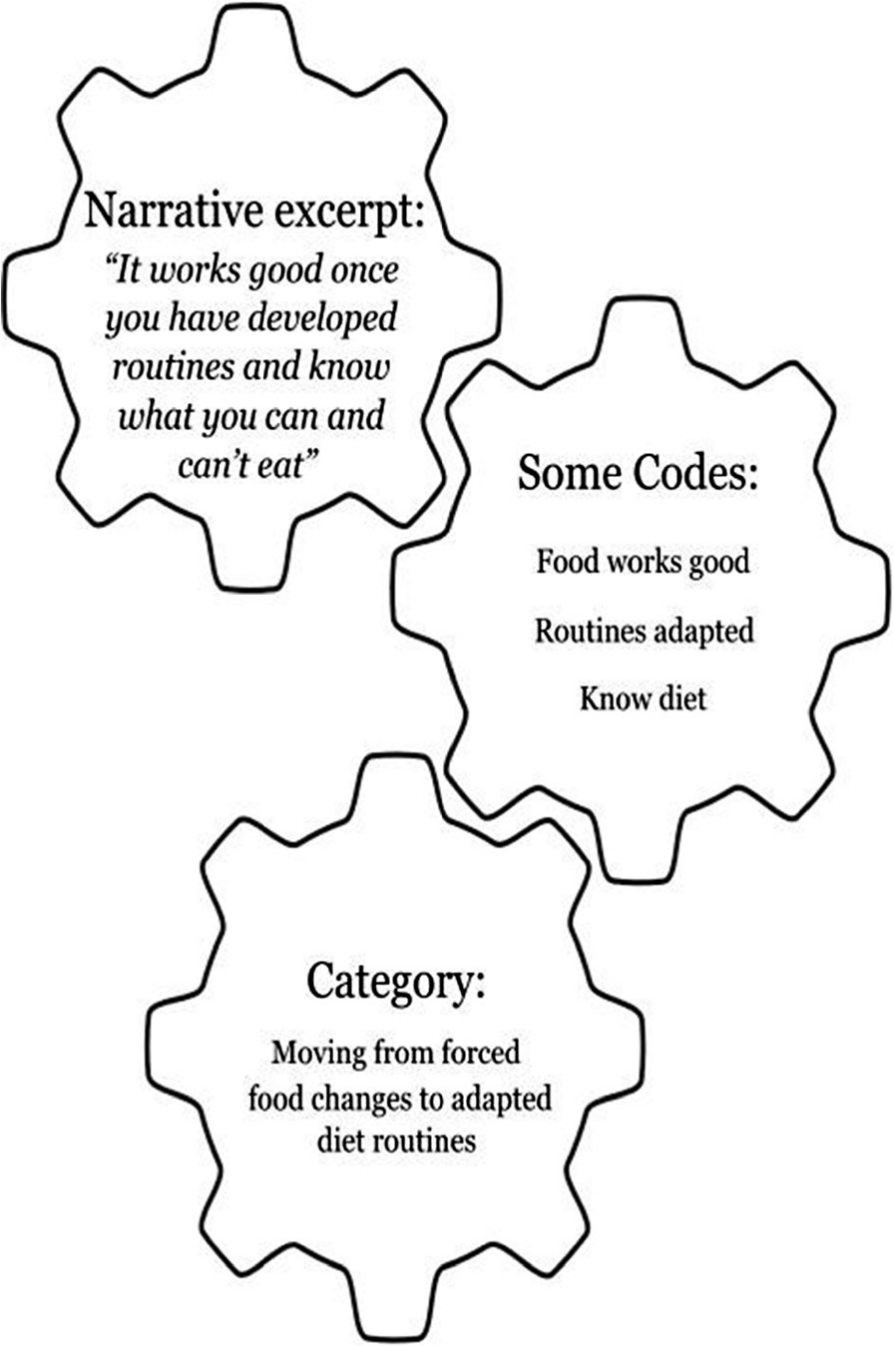
**Process of moving from the text, to codes, to category in the qualitative content analysis.** Detailed legend: A qualitative content analysis approach was used to analyze the narratives and gain a deeper understanding of content while remaining close to the text.

The narratives ranged from a few lines to a few pages. The narratives were transcribed verbatim and entered into OpenCode software [46] where they were grouped according to region and if the narrative was from the one- or five-year follow-up. In order to elucidate the adolescents’ long-term experience, the analysis focused specifically on comparing the one- and five-year experiences and interpreting whether there was an evolution and what was unique after five years. A qualitative content analysis approach, aimed at gaining a deeper understanding of the content while remaining close to the text and further interpretation an abstract level [47], was utilized for the analysis. In the narratives written by these adolescents we found that the texts were concise and were coded directly from the original texts. A concept map was created to visualize linkages of the codes and to cluster them into final categories, which represented the dominant manifest content (Figure 2). Finally, while constantly referring back to the narratives, codes, clusters, and categories, we interpreted the abstract meaning that describes the overall experience of these adolescents and is presented by the overall theme.

## Results

The narratives written by these screening-detected adolescents reflected a variety of experiences both at the one- and five-year follow-ups. By focusing on interpreting how the experiences had evolved and were unique in the five-year follow-up, we found that there was maintenance and evolution in the perceptions, practices, and beliefs after five years of living with screening-detected CD.

**Figure 3:**
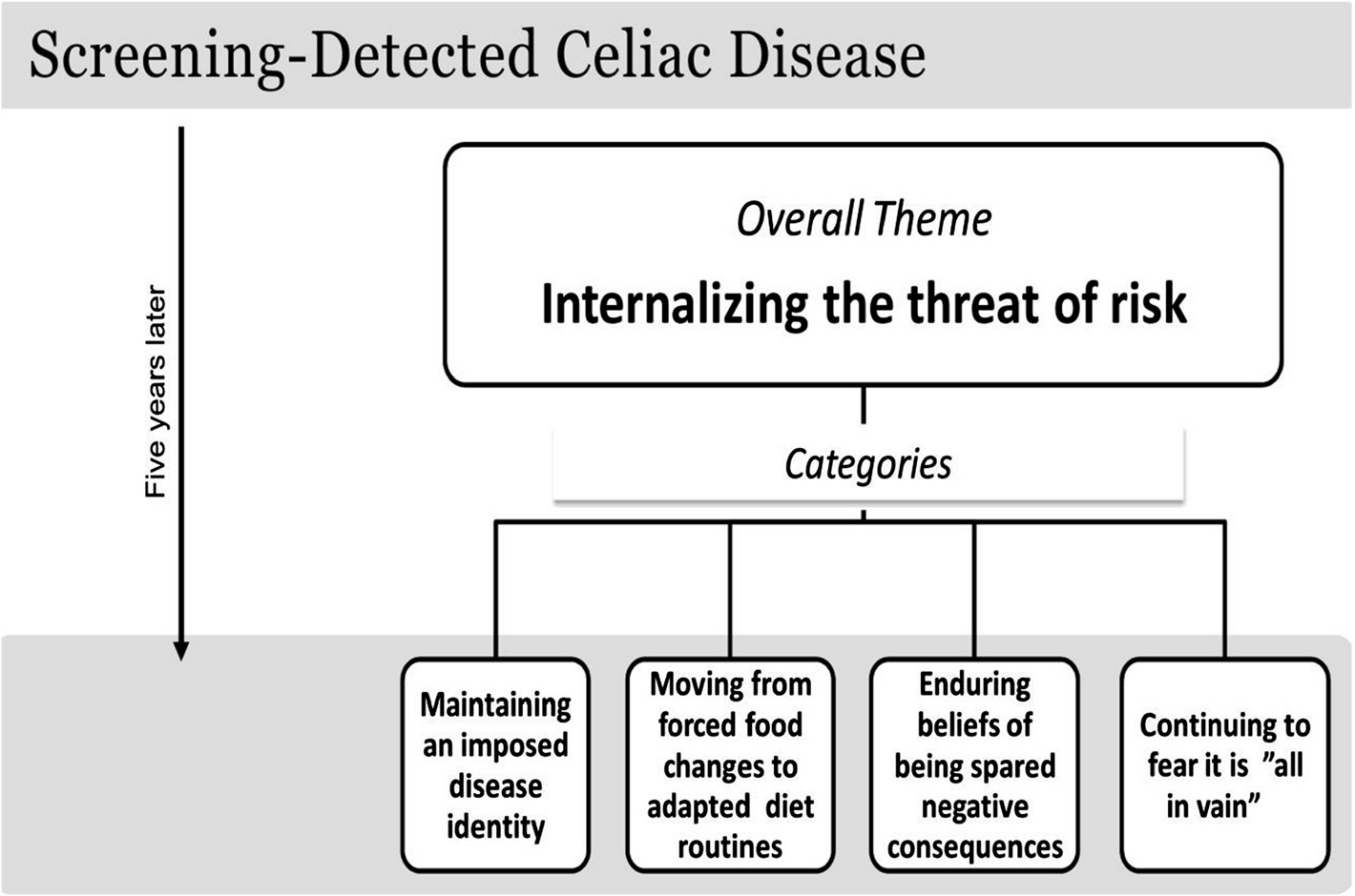
**Evolution in perceptions, practices, and beliefs of adolescents five years after screening-detected celiac disease diagnosis.** Detailed legend: The overall theme – *“Internalizing the threat of risk”* – is supported by four categories: “Maintaining an imposed disease identity”, “Moving from forced food changes to adapted diet routines”, “Enduring beliefs of being spared negative consequences”, and “Continuing to fear it is “all in vain”.

The overall theme **–*“Internalizing the threat of risk”*–** illustrates that being detected through screening and the internalization of the threat of risk for future health complications have impacted how these adolescences felt about the diagnosis, coped with the GFD, and thought about CD screening (Figure 3)*.* The theme is supported by four categories: **“Maintaining an imposed disease identity”** describes how these adolescents continued to define themselves in relation to the screening. **“Moving from forced food changes to adapted diet routines”** illustrates how after living with CD for five years they described habits, routines, coping strategies, and the financial burden of the GFD. The category **“Enduring beliefs of being spared negative consequences”** illustrates that they still believed that being diagnosed and following the GFD had spared them from negative health consequences, or would do so in the future. Some were still concerned that the diagnosis and diet might not be beneficial, and this is illustrated with the category, **“Continuing to fear it is “all in vain”**. In the following text we present the categories including some codes within those categories and examples of excerpts from the five-year narratives that support the interpretation of the dominant issues and also demonstrate variation.

### Maintaining an imposed disease identity

The adolescents’ descriptions of their feelings when diagnosed were similar in the one- and five-year follow-ups. They still related how they feel now to when they were diagnosed. Their reactions to news of the diagnosis varied and some examples of codes clustered in this category include: unfortunate, shock, surprise, sad, happy, suspected, exciting, miserable, unfortunate, not a big deal, accepted, rejected, ruined life, relieved, stigma, neglected, supported, alone, pressure to feel better, school better now, etc.

*“To be the only one in the whole school who is “different” was among the worst things that I have had happen to me”* (girl)

*“I felt confused and disappointed because my parents had forced me to be with the study”* (girl)

*“..relief to find out that it was GI that was the problem because I had felt bad before I found out”* (boy)

*“Since then my life has improved considerably. I am more energetic and have more energy to do more”* (girl)

*“Surprised, because I hadn’t experienced any big problems that I could connect to CD”* (boy)

When they described how it was to live with CD they wrote about how it was in school, while eating out, at home, and away from home. They described a variety of experiences indicating how they felt supported or unsupported by others.

“*Unfortunate in social situations otherwise intolerance isn’t something that disturbs me”* (boy)

*“I usually say that you become “food handicapped”* (girl)

*“I have to plan more when I go away. Always remember to inform that I don’t eat gluten”* (girl)

*“The whole family had to change how they think, we became more conscious of what was in the food we eat”* (girl)

After five years, they had had the chance to meet new people and be in new environments as a person with CD that was identified as having CD from the beginning. This resulted in a more comfortable scenario compared to when then had to explain changes in diet directly after being diagnosed.

*“I think it would be good to test all but at a much younger age, maybe the year before you start school so they will see it is obvious that you eat gluten-free food”* (girl)

The girls were more prone than the boys to describe the overall experience as negative while the boys gave the impression that adjusting to the diagnosis and diet was not very remarkable.

*“Life after was also so and so, it felt like I was a burden for everyone”* (girl)

*“It was difficult then, but that was long ago”* (boy)

### Moving from forced food changes to adapted diet routines

There were a variety of experiences with the GFD. They felt supported, neglected, and stigmatized. They faced internal and external struggles and were forced to cope. Although they still wrote about characteristics of food and certain environments, after five years they also referred to new habits and routines, which represented a new way of reflecting. Codes included in this category included: bad, boring, no big deal, just exclude gluten, tastes bad, alternatives available, force to change, forced to plan all the time, must think all the time, adapted, learned, crave food, accept, tricky, burden, difficult when out, responsibility, special food, expensive, difficult to bake, etc.

*“In the beginning it was difficult, I didn’t know what I could eat, but now it feels natural to have CD”* (boy)

Another evolution that we saw after five years involved increased personal responsibility for the diet reflected in the adaption of new habits and because there were accounts of the financial burden imposed by the diet.

*“It became more for me to take responsibility for”* (girl)

*“The biggest change is with my economy”* (girl)

Although there were similarities in the boys’ and girls’ stories, the girls were more likely to describe the struggle to adapt and to describe the financial burden of the GFD.

*“Everything was different because you must think every time you eat, the food is expensive, and it is harder to eat out”* (girl)

### Enduring beliefs of being spared negative consequences

The threat of complications related to CD and not following the GFD impacted how they lived with CD and the beliefs they had about screening all children for CD. The accounts described following the diet to avoid developing complications later on. They also gave this as a reason why everyone should be screened. Codes include in this category included: felt benefit, long-term consequences exist, promised benefit motivates, test all because there is benefit, etc.

*“Sometimes I regret that I was tested-but it was for the best”* (boy)

*“I didn’t suffer from eating flour but I definitely think that everyone should be tested, you don’t want to go around being “sick”* (girl)

*“I think all kids should be tested for the simple reason that my life has improved so much…”* (girl)

Both boys and girls thought all children should be tested at an earlier age, and that this should be done to avoid future complications or have an easier time adapting to the GFD.

*“Kids should be tested at an early age, anyway before puberty so that their growth isn’t effected badly, or so that it effects development as little as possible and doesn’t harm the body”* (girl)

### Continuing to fear it is “all in vain”

Even after five years some children conveyed doubts as to whether they really had the disease. They questioned the benefit of being diagnosed and expressed worry and regret about getting the diagnosis. Some said that they sometimes followed the GFD while others said they quit. Some codes in this category included: quit diet, no benefit, not sure benefit, no change, question benefit, sometimes regret, blissfully ignorant, didn’t suffer from it, unsure if long-term consequences exist, test only symptomatic, etc.

*“It felt difficult to find out and I have many times thought that it would have been better to be blissfully ignorant”* (girl)

*“I don’t think all kids should be tested, I think this study is horrible because I didn’t feel anything, but I think those that feel bad should test themselves”* (girl)

*“For the last 3 months I have eaten regular food to see how the tests will be”* (boy)

Overall, they described a willingness to follow the diet, although the girls were more prone to have quit or have said they were not strictly compliant because they had not noticed any benefit.

*“I don’t feel a big difference if I eat gluten or not which has led to me following it (the diet) bad”* (girl)

## Discussion

One year after diagnosis some these screening-detected adolescents also completed questionnaires (n = 93) and 54% reported feeling better, 4% worse, 37% no different, and 5% did not remember how they felt before diagnosis [43]. What we discover from their narratives, written one and five years after diagnosis, is that being screening-detected and the length of time they have lived with CD has impacted their perception of risk and how they have integrated the disease into their lives. Some have adjusted to the disease and adapted new habits and coping strategies to deal with the GFD. Others still doubt they have CD or that being detected has benefited them.

### Threat of risk

We found that the perception of risk by these adolescents had been impacted by participating in and receiving the CD diagnosis through screening. Being detected through a population-based CD screening study and the threat of future health complications impacted how these screening-detected adolescents felt about the diagnosis, coped with the GFD, and thought about CD screening even five years after diagnosis. Avoiding possible negative consequences was the motivation for following the GFD and these adolescents generally believed that by being detected they were saved from imminent negative consequences. This belief has been discussed as a strategy to deal with disease and screening-based diagnoses to feel good about being detected and having to cope with disease and treatment [22,48,49]. However, there were also adolescents who even after five years doubted the accuracy and benefits of the diagnosis.

### Living with CD

Living with a chronic disease can pose special challenges in adolescence, when many life transitions are being integrated into the young person’s identity and sense of self [50]. The screening-detected adolescents experienced stigmatizing feelings related to the diet. They described social gatherings as constant situations when exposure and visibility are high. Not eating, trying to find an alternative, or bringing their own snack can be problematic and draw unwanted attention. Clinically-detected adolescents have also reported that questions about their diet and disease are experienced as demanding [51]. Treatment with a GFD can be considered costly, complex, and impacts all activities involving food [52]. The adolescents in this study mostly described living with CD as manageable, although there were also some who said that being diagnosed was terrible and some who quit the diet. The adolescents in this study experienced unwanted attention related to the GFD, but had also adjusted new habits and coping strategies. When adults were asked to recall emotions after CD diagnosis in a Canadian survey, relief was the most common emotion, but these feelings decreased after starting the diet [52]. Respondents reported feeling frustrated, overwhelmed, and isolated, demonstrating that having to make lifelong changes in dietary patterns can have significant emotional impact [52]. Difficulties and negative emotions were experienced less frequently by those who had been on the diet for more than five years, although food labeling and eating away from home remained problematic [52]. Other studies have shown that the diet can be a burden even after being on it for several years [53-55], and our findings confirm this. Similar to what has previously been reported; we found that limited availability of gluten-free alternatives, lack of symptoms, and negative feelings can pose a challenge to compliance [51,56]. Studies in which compliance with the diet has been considered have shown rates for strict adherence ranging from 42% to 96%, depending on definitions and methods of assessment [57-60]. It has been suggested that those who are detected through screening can be at particular risk of poor adherence [61]. However, some of these screening-detected adolescents also answered questionnaires one year after diagnosis and 72% reported always eating gluten-free [43]. We found that after one year with the disease they wrote about the characteristics of the food and described how they were always forced to think about food and plan what to eat. After five years they described their food habits and routines and gave examples of strategies, such as having a separate food storage area or their own butter knife.

### Boys and girls

Boys and girls overall had similar experiences, but with some different patterns. The girls were more likely to describe the overall experience as negative and describe feeling like they were a burden to others. Other studies with adult women with CD have reported similar findings. One study found that adult women with CD experienced emotional distress related to the GFD more than men [52]. Women with CD have also been shown to experience the disease as more of a burden than men [55,62]. The boys in this study wrote about the adjustment to the diagnosis and diet as something they just did. Boys gave examples of changes they made, while girls described the struggle to adapt, the financial burden, or that they quit the diet when they did not experience any benefit. Others have found that men with CD used problem-orientated methods to cope, while women sought emotionally oriented strategies and ultimately showed less satisfaction with the outcome and more distress due to the daily restrictions in their lives [55]. Taking into account that females and males cope differently with disease and lifestyle changes suggests the need to individualize support for those with CD.

### Strengths and limitations

Our results are unique because they are reflective of those living with unrecognized CD detected through a population-based screening study. Most studies that assess the effects of being diagnosed involve individuals who were tested clinically because of symptoms or because they were considered at high risk for CD. Individuals diagnosed clinically after presenting with symptoms or because they are high risk will have a different experience than those found through mass screening [63]. All the adolescents who were diagnosed as a result of the ETICS screening study at the time this study was planned were invited to participate and the narratives are reflective of their interpretation of the experience. Although we can apply what we have learned in relation to this context, we cannot assume that the experience would be the same when being diagnosed through any screening. To increase validity and ensure that the results were grounded in the narratives, there was continuous comparison of codes, categories, and the theme back to the narratives. To increase the study’s trustworthiness the researchers were from varying disciplines and contributed with expertise in CD research, pediatric medicine, qualitative research, and nursing. The data was rich enough to capture variation, but it is important to consider the nonparticipants. It could be that those who declined to participate were more likely to have rejected or ignored the diagnosis or quit the diet. Their stories may have led a deeper understanding of the challenges of living with screening-detected CD.

## Conclusions

There was both maintenance and evolution in the perceptions, practices, and beliefs of the adolescents after five years. Some have adjusted to the disease and adapted new habits and coping strategies to deal with the GFD, while others still doubt they have CD or that being detected was beneficial. The transition to adapting to the disease and the diet is an ongoing process and the experiences differ among the adolescents and between the girls and boys, illustrating the importance of providing ongoing and individualized support for those with screening-detected CD as they adjust to this chronic disease and the GFD.

## Abbreviations

CD: Celiac disease; GFD: Gluten-free diet; HRQoL: Health-related quality of life; QOL: Quality of life.

## Competing interests

The authors have no competing interests or financial relationships relevant to this article to disclose.

## Authors’ contributions

KN (PhD, RN) contributed to the conception and design of the study, performed the analysis, and wrote and revised the manuscript. AR (PhD, MD) contributed to the conception, design, and data collection and critically reviewed and gave feedback during drafting and revising of the manuscript, and approved the final manuscript as submitted. ME (Prof.) contributed to the conception, design, data collection and analysis and critically reviewed and gave feedback during drafting and revising of the manuscript, and approved the final manuscript as submitted. AI (Assoc. Prof., Pediatrician) contributed to the conception, design, and data collection, critically reviewed and gave feedback during drafting and revising of the manuscript, and approved the final manuscript as submitted.
